# Case of Abscess of Parotid Gland

**Published:** 1860-05

**Authors:** J. Lykins

**Affiliations:** Kansas City, Mo.


### CASE OF ABSCESS OF PAROTID GLAND,

REPORTED BY J. LYKINS, M. D., OF KANSAS CITY, MO.

In August, 185–, Mr. J. J. a respectable farmer, aged 45, was attacked with congestive fever, ushered in by a chill. Upon the invasion of second chill, I was called to see the patient. Found the extremities cold, pulse hardly perceptible, patient unconscious, passing involuntary stools, etc. By the most prompt and energetic treatment, after three hours effort, pulse rose and fever set up.

2nd day.—Kept patient under the influence of free portions of quinine, remained with him three or four hours, preceding and during the time of expected chill; patient escaped both chill and fever, and was dismissed as requiring no further treatment.

4th day, 4 o'clock, P. M.—Was called in great haste to the patient, found him suffering most intense pain, and upon examination found its seat to be the Parotid Gland, which proved to be slightly enlarged and too painful to be touched. The patient having been subjected to a vigorous treatment of Sub. Mur. Hydrg. and Sulph. Quin. in order to break up the fever, was put upon the use of saline cathartics and the usual lotions externally.

6th day.—Jaws fixed, gland greatly enlarged, general tumefaction much increased, jaws too rigid to admit of anything entering the mouth except fluids; patient delirious. Prescribed castor oil to be followed by quinine, external applications the same.

7th, 8th, and 9th days.—Treatment continued, left eye closed by swelling, articulation suspended.

10th and 11th days.—Prognosis hopelessly unfavorable; called in Drs. Boggs & Caldwell, both of whom agreed without hesitation, in pronouncing the case one of Abscess of the Parotid Gland, supervening upon the congestive stages through which the patient had passed during the first attack.

12th and 13th days.—Tumor purple; first indications of pus deeply seated.

14th day.—Minute portions of purulent matter escaping from the ear, with indications of pus pointing over the centre of the gland.

15th day.—Introduced lancet, pus was reached easily, but escaped slowly.

16th day.—Patient feeble, discharging enormous quantity of dark, morbid bile, from the bowels; no relaxation of the jaws; treatment continued.

17th day.—Plunged lancet into the tumor, anterior to the ramus of the lower jaw, below the masseter muscle; as before reached the pus, but it would not flow. Flax-seed meal poultice applied.

18th, 19th, and 20th days.—Pus tenacious, escaping slowly, skin sloughed away below the ear to the angle of the jaw, exposing viscid pus adherent by cellular tissue, and resembling end of grass rope.

21st and 22d days.—Jaws slightly relaxed, swelling abated partially, patient desires food, is more rational.

23rd, 24th and 25th days.—Slough enlarged, discharge exceedingly offensive.

26th, 27th and 28th days.—Takes soup freely, and desires meat, improving; tonics continued.

29th, 30th and 31st days.—Opening under the ear enlarging pendant from which hangs the suppurating inferior portion of the Parotid Gland. Attempted to cut it away with Surgeons' scissors; so resistant as to cause the scissor blades to chew; left side of the face paralysed, and cheek drawn aside.

32nd and 33rd days.—Patient rapidly improving, jaws are partially moveable; suceeded in removing a portion of destroyed Gland, which brought away with it the trunk of the Portio dura and its branches Temporo facial and cervico facial, the external carotid artery, etc.

34th day.—Patient continues to improve; Gland slowly coming away. This continued many weeks; at last the ulcer healed, and the patient regained his health.

Happily for us this patient now resides near our city, and to an examinaton of the cicatrix, and to the evident absence of the Parotid Gland, I beg leave to invite the attention of the Editors of this Medical Journal. I do this with the more interest as Dr. Titus Deville in the Chicago *Medical Examiner* has denied that the Parotid Gland is liable to disease, and has thereby in my opinion, convicted himself of the ignorance which he has charged upon Prof. Brainard.

Dr. Caldwell of Independence, a most intelligent Physician at the time of the above related case, informed me that he had in this country witnessed two cases of abscess of Parotid Gland, consequent upon congestive fever, both of which proved fatal.—*Kansas City Med. and Surg. Review.*

